# The Prognostic Significance of Wnt-5a Expression in Primary Breast Cancer Is Extended to Premenopausal Women

**DOI:** 10.1371/journal.pone.0070890

**Published:** 2013-08-22

**Authors:** Janna Sand-Dejmek, Roy Ehrnström, Pontus Berglund, Tommy Andersson, Lisa Ryden

**Affiliations:** 1 Experimental Pathology, Department of Laboratory Medicine, Lund University, Skåne University Hospital, Malmö, Sweden; 2 Surgery, Department of Clinical Sciences Malmö, Lund University, Skåne University Hospital, Malmö, Sweden; 3 Clinical Pathology, Department of Laboratory Medicine Malmö, Lund University, Skåne University Hospital, Malmö, Sweden; 4 Surgery, Department of Clinical Sciences Lund, Lund University, Skåne University Hospital, Lund, Sweden; Health Canada and University of Ottawa, Canada

## Abstract

Wnt-5a protein expression in primary tumors from unselected breast cancer patients has revealed a tumor suppressive function of the protein. However, *in vitro* experiments on human breast cancer cells have reported contradictory results, indicating both a tumor suppressive and promoting functions of Wnt-5a. This could be due to various functions of Wnt-5a in different subgroups of patients. The unselected cohorts analyzed to date for Wnt-5a protein expression contained few premenopausal patients. The aim of the present investigation was to evaluate the prognostic significance of Wnt-5a protein expression in a cohort of premenopausal women with comprehensive data on biomarkers, molecular subtypes and long-term outcome. In a randomized trial of adjuvant tamoxifen versus no adjuvant treatment, 564 premenopausal primary breast cancer patients were included. The median follow-up time was 14 years. A tumor tissue array was constructed and 361 samples were evaluated for Wnt-5a reactivity by immunohistochemistry. The primary end-point was recurrence-free survival. Wnt-5a protein expression was reduced or lost in 146/361 of tumors and correlated to younger age, estrogen receptor (ER) negativity and triple-negative phenotype. Wnt-5a was a prognostic factor in the whole cohort (*p* = 0.003). In patients with ER-positive tumors, Wnt-5a was an independent positive prognostic marker (HR 0.51 95% CI: 0.33–0.78 *p* = 0.002) and HER2 a negative prognostic marker (HR 2.84 95% CI: 1.51–5.31, *p* = 0.001) in a Cox multivariate analysis adjusted for standard prognostic markers and tamoxifen treatment. In the ER-negative subset, Wnt-5a added no prognostic information. In a subgroup analysis, Wnt-5a was significantly associated with better prognosis in patients with Luminal A tumors (*p* = 0.04). Conclusively, our results suggest that loss of Wnt-5a is a valuable prognostic marker in premenopausal breast cancer patients in particular in patients with ER-positive tumors and out-performed conventional prognostic factors in this subset of patients.

## Introduction

Breast cancer is a heterogenous disease. The heterogeneity of the disease affects not only the prognosis but also the choice of treatment [1]. One very important factor in this context is the menopausal status of the patient. Breast cancer in premenopausal women is generally more aggressive and has a poor prognosis [1]. As a reflection of the difference between breast cancer in women with varying menopausal status, breast cancer in premenopausal females are more often estrogen receptor negative (ER−) although >50% of premenopausal breast cancer are ER+ [2]. Furthermore, premenopausal breast cancers usually have higher proliferation indices than tumors in postmenopausal women [3]. Consequently, studies on the prognostic value of new biomarkers have to properly address this heterogeneous property of breast cancer.

Wnt proteins belong to a family of secreted proteins involved in a wide range of cellular processes. Wnt signaling can be broadly divided into two categories; the canonical, ß-catenin-dependent pathway and the non-canonical ß-catenin-independent pathway. Wnt signaling is initiated by binding of a Wnt ligand to its receptor(s). Canonical Wnt signaling results in stabilization of the key transcription factor ß-catenin, leading to its translocation into the nucleus where it drives the expression of target genes such as cyclin D1 and c-MYC [4]. Wnt-5a is a non-canonical Wnt ligand that is ubiquitously expressed in normal tissues [5]. In cancer, Wnt-5a is often dysregulated and the protein has been implicated both in tumor suppressive as well as in tumor promoting activities [6]. In good agreement with these findings, Wnt-5a has been recognized both as a marker of favorable and of poor outcome in primary cancers. We, and others, have previously shown that loss or low expression of Wnt-5a in the primary tumor has an unfavorable prognostic value in breast, prostate and colon cancer [7], [8], [9], [10]. Loss or reduced Wnt-5a expression has also been reported in liver metastases from patients with colorectal cancer [11]. In primary hepatocellular carcinoma, neuroblastoma, lymphoma and thyroid cancer, loss of or reduced Wnt-5a expression has also been associated with an adverse outcome [6], [12]. On the other hand, a tumor promoting function has been strongly documented for Wnt-5a in melanoma and in melanoma elevated Wnt-5a expression has also been associated with poor prognosis [13], [14]. Similar results were also reported for the role and prognostic properties of Wnt-5a in gastric cancer [15]. Collectively, these findings underscore the complex functional and prognostic roles of Wnt-5a in cancer.

In experimental studies, we found that reconstitution of Wnt-5a signaling decreased the migratory capacity and invasiveness of cultured breast cancer cells and that administration of a Wnt-5a-mimicking peptide significantly reduced breast cancer metastases in a mouse model [16], [17]. Recently these findings have been confirmed and in addition it was shown that when 4T1 breast cancer cells were transfected to express Wnt-5a their injection into the tail vein of Balb/c mice resulted in significantly less lung metastases as compared to control cells [18]. Despite all these findings it has also been reported from *in vitro* experiments that Wnt-5a can promote migration and invasion of breast cancer cell lines [19]. It is possible that these contradictory results could be due to different properties of the cell lines investigated and that this might reflect different functional properties of Wnt-5a in the different subgroups of breast cancer. Such a possibility is strengthened by the fact that in the unselected cohorts analysed for Wnt5a protein expression there were few premenopausal breast cancer patients [7], [10].

Here, we investigate for the first time the prognostic value of Wnt-5a expression in breast cancer tissue from a large cohort of premenopausal patients with comprehensive data on molecular subtypes and long-term outcome.

## Materials and Methods

### Ethics Statement

Verbal informed consent was provided from all included patients. At the time being, written informed consent was not mandatory. The study (SBII:2) and consent procedure were approved by the regional ethical committees at the Universities of Lund and Linköping. Documentation of verbal informed consent for included patients as well as randomization was performed at the Regional Oncological Centers.

### Clinical trial

**Figure 1:**
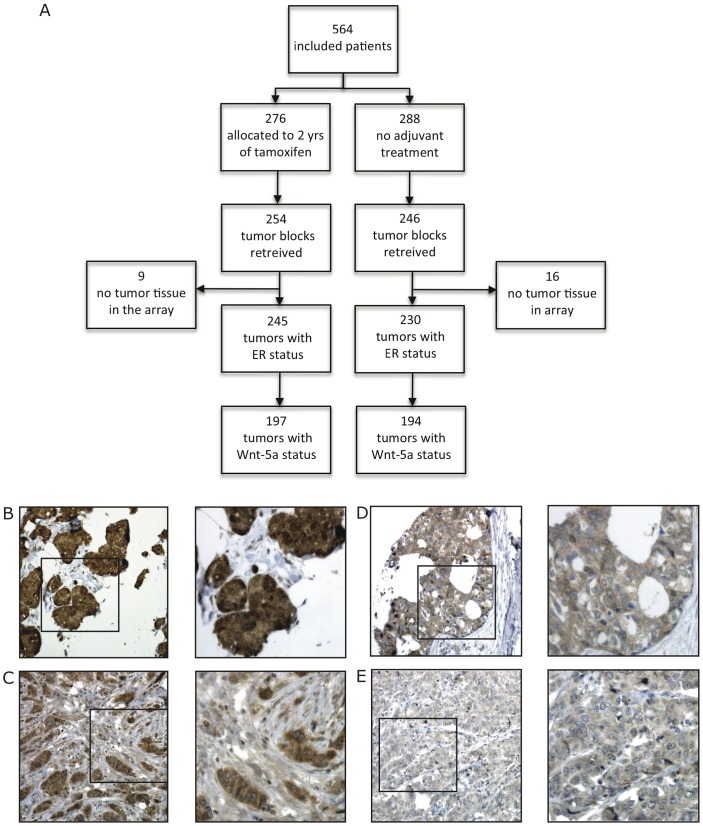
Design of clinical trial and immunoreactivity for Wnt-5a. A. Flow chart defining patients enrolled in clinical trial. B–E. Immunoreactivity for Wnt-5a protein in representative sections of invasive breast carcinomas. B. Tumor with strong (+++) Wnt-5a staining, C. Moderate (++) staining for Wnt-5a, D. Weak (+) staining for Wnt-5a, and E. No (−) Wnt-5a expression (left hand panels: 10× magnification; right hand panels: 40×).

Premenopausal patients diagnosed with stage II primary breast cancer (n = 564) between January 1984 and September 1991 were enrolled in a randomized controlled multi-center trial comparing two years of adjuvant tamoxifen (TAM) with no adjuvant treatment. A flow-chart of the study is shown in Figure 1. Patients were included irrespective of hormone receptor status and less than 2% of the included patients received additional systemic adjuvant therapy. Tumor blocks could be retrieved from 500 of the 564 randomized patients and a tissue microarray (TMA) was constructed (see below). ER status was determined in 475 of the tumors. The trial design, primary treatment and clinical outcome in relation to treatment arm have been described in detail before and information on age, tumor size, lymph node status and Nottingham Histological Grade (NHG) was available [20]. Recurrence-free survival was the primary end-point in the study and the median follow-up time was 13.9 years for patients alive and free of breast cancer-related events. The study was approved by the Ethical Committees at the University of Lund the University of Linköping. Randomization was performed by the Regional Oncological Center and informed consent was registered for all included patients. The study has been included in the meta-analysis by the Early Breast Cancer Trialists'Collaborative Group [21].

### Tumor Tissue Microarray and immunohistochemistry

Areas representative of invasive cancer were marked on the haematoxylin and eosin stained slides and two separate tissue microarrays were constructed, one using a manual (MTA-1) and the other an automated (ATA-27) arrayer (both from Beecher Inc, Sun Prairie, Wisconsin, USA). Two 0.6 mm tissue cores were taken from each donor block and mounted in a recipient block. There were approximately 200 tissue cores in each recipient block. Cores were generally taken from the peripheral part of the tumor in cases where the tumor had relatively well defined borders. For technical reasons, in more diffusely growing tumors, areas with the highest tumor cell density were primarily targeted. Sections (4 mm thick) were dried, dewaxed, rehydrated, and microwave treated for 5 minutes in citrate buffer (pH 6.0) before being processed in an automatic immunohistochemistry staining-machine (Techmate 500; Dako, Copenhagen, Denmark) using antibodies against Wnt-5a (dilution 1∶200) or Ki-67 (1∶200, M7240; DAKO). The polyclonal antibody against Wnt-5a was developed and characterized in our laboratory [22], [23]. For antibodies against ER, PR, VEGF-A, and HER2, heat-mediated antigen retrieval was performed using microwave treatment for 2×5 minutes in citrate buffer before being processed either in the Ventana Benchmark system (Ventana Medical Systems Inc., Tucson, AZ) using pre-diluted antibodies to ER (anti-ER, clone 6F11), PR (anti-PR, clone 16) and HER2 (Pathway CB-USA, 760–2694), or in the Techmate system (TechMate500, DAKO, Denmark) for VEGF-A using a polyclonal antibody (clone A-20, Santa Cruz, CA, USA). Cytoplasmic staining of Wnt-5a was assessed by two investigators, one of whom is a board-certified pathologist (RE), according to intensity (negative  = 0, weak  = 1, moderate  = 2, strong  = 3). No Wnt-5a staining was present in the nucleus. Cores with discordant scores were reassessed jointly and a consensus was reached. For immunohistochemical evaluation of Ki-67, a scoring system based on the estimated fractions of positively staining nuclei was used as follows: 0, 0–1%; 1, 1–10%; 2, 11–25%; 3, 26–50%; and 4, 51–100%. The intensity of the nuclear staining for Ki-67 varied slightly, but was distinct in most cases. For statistical evaluation, tumors were classified as I = 0–10% Ki67 positive nuclei; II = 11–25% positive nuclei, and III  = >25% positive nuclei. ER-negativity and PR-negativity was defined as <10% positively staining nuclei, according to current clinical guidelines in Sweden. Staining of VEGF-A was only evaluated in invasive tumor cells and evaluated according to a semi-quantitative scale for staining intensity (0–3). In the further analysis absent and weak staining intensity was considered as low protein expression and moderate and intense staining was considered as high protein expression. HER2 was assessed by IHC and fluorescence *in situ* hybridization (FISH) as previously described [24]. Briefly, HER positive (HER+) tumors were defined as amplified and/or 3+ by IHC, HER negative (HER−) tumors as non-amplified or 0–2+ by IHC.

### Subgroup classification

The categorization of subtypes was made using the St Gallen International Breast Cancer Conference 2011 criteria and modified to the present a cut-off for Ki67 at 25% [3]: Luminal A (ER+ and/or PR+, Ki67<25% and HER2−), Luminal B (ER+ and/or PR+, Ki67>25% and/or HER2 +/−), HER2-type (ER−, PR− and HER2+), Triple-negative type (ER−, PR− and HER2−).

### Statistical analysis

Recurrence-free survival (RFS) was the primary endpoint and all analyses were done according to the intention to treat rule. Recurrence-free survival included local, regional and distant recurrences and breast-cancer death as primary events. Comparisons of clinical data and tumor characteristics according to Wnt-5a-status were evaluated by Chi-square -test and by Chi-square-test for trends for variables with more than two categories. Kaplan-Meier curves were used to illustrate survival according to Wnt-5a expression and log rank test to assess for equality of survival curves. Hazard ratios were estimated using Cox proportional hazards model for RFS in uni- and multivariate analyses. The model was used to estimate the interaction between TAM treatment and Wnt-5a expression measuring a possible difference in treatment effect depending on Wnt-5a expression and an interaction variable was constructed (TAM treatment +/− x Wnt-5a expression +/−). Calculations were performed using SPSS version 19.0 (SPSS Inc., Chicago, IL). All *p*-values corresponded to two-sided tests and values of less than 0.05 were considered significant. The presented *p*-values have not been adjusted for multiple testing.

### Cell culture and transfections

MDA-MB-231 cells (obtained from ATCC) were kept at 37°C in a humidified atmosphere of 5% CO_2_ and cultured in DMEM medium supplemented with 10% fetal bovine serum, 5 U/ml penicillin, 0.5 U/ml streptomycin and 2 mM glutamine. Cells were seeded in 6-well plates and allowed to grow for 24 hours before transfection with either 1 µg pcDNA3 (empty vector) or 1 μg ERα plasmid. Transfections were carried out using Lipofectamine 2000 (Invitrogen) according to the manufacturer's recommendations. Seventy-two hours post-transfection, cells were used in invasion assays or harvested for protein extraction.

### Western blotting

Cells were lysed in lysis buffer (20 mM TRIS-HCl pH 7.5, 150 mM NaCl, 30 mM Sodium Pyrophosphate, 1 mM EDTA, 1.5 mM MgCl_2_, 0.2 mM Sodium Orthovanadate, 10% Glycerol, 1% Triton X-100 and 1 tablet of Complete-Mini protease inhibitor (Roche)/10 ml lysis buffer) for 30 min on ice and centrifuged for 30 min, 14.000 rpm at 4°C. Protein lysates were subjected to SDS-PAGE and transferred to PVDF membranes. ERα expression was detected by probing with an antibody against ERα (sc-7202, Santa Cruz, CA) diluted 1∶500. Equal loading was confirmed by probing with an HRP-conjugated antibody against ß-actin (ab20272, Abcam, MA) diluted 1∶10.000. Protein bands were detected using the Immobilon Western Chemiluminescent HRP Substrate (Millipore, MA). Protein lysate from the ERα-positive breast cancer cell line T47D was used as a positive control.

### Invasion assay

Invasion assays were carried out using Matrigel invasion chambers (BD Franklin Lakes, NJ) with 8 μm pore size in a 24-well plate format. Seventy-two hours post-transfection cells were serum starved for 6 hours, detached using Versene (Gibco) and resuspended in serum-free medium. Subsequently, 500 μl serum-free medium containing 25 000 cells was added to the upper chamber. Medium containing 10% serum was added to the lower chamber. For treated samples, recombinant Wnt-5a (0.2 μg/ml) or the Wnt-5a mimicking peptide Foxy5 (100 nM) was added to the cell suspensions [16]. The cells were then allowed to invade. After 20 hours, the upward facing side of the membrane was wiped to remove the Matrigel. Invading cells at the downward facing side were fixed in 4% paraformaldehyde, stained with crystal violet and counted. The invasion assay was repeated seven times in duplicates and the result is presented as means relative to the untreated controls and SEM. Statistically significant differences were assessed with two-tailed, paired *t*-test (**, P<0.01; ***, P<0.001).

## Results

### Immunohistochemical expression of Wnt-5a in premenopausal invasive breast cancer

In the current study, Wnt-5a expression was evaluable for 391 cases. Figure 1A shows a flow chart defining patients enrolled in the clinical trial (Figure 1A). To avoid bias due to heterogeneous Wnt-5a expression, only cases with 2 or more evaluable cores on the TMA were included, hence 30 cases with only one available core were excluded. When present, Wnt-5a was expressed in the majority of tumor cells (>75%) and therefore only staining intensity was included in the statistical analyses. One hundred and fifty-six (40.4%) tumors exhibited complete lack of, or very low Wnt-5a expression (Wnt-5a = 0 or 1), and 215 tumors showed moderate or strong Wnt-5a expression (59.6%, Wnt-5a = 2 or 3). Figures 1B–E shows tumors with different staining intensities, ranging from negative to strong staining. Staining was exclusively cytoplasmic, with no tumors exhibiting nuclear or membranous staining (Figures 1B–E).

### Correlation between Wnt-5a expression and clinico-pathological variables

Wnt-5a protein expression data were dichotomised into absent ( = 0) or low ( = 1) staining versus moderate ( = 2) or strong ( = 3) staining. As shown in Table 1, loss of Wnt-5a was associated with younger age (p = 0.016), ER negativity (p = 0.016) and TNB- phenotype (p = 0.04). The correlation between Wnt-5a and ER is well in line with our previously published data [7] and the finding isn't surprising, given that a majority of well established prognostic factors such as Ki67, NHG and HER2, show the same expression pattern in relation to that of ER. We also noted a positive association between Wnt-5a expression and VEGF-A expression, in accordance with previous findings in prostate cancer (25). No association was found between Wnt-5a expression and lymph node status, tumor size, histological tumor grade, or proliferation index (Ki67). Tumor characteristics did not differ between tumors with and without Wnt-5a status, confirming that the evaluated tumors were representative of the whole cohort (not shown).

**Table 1:** Associations between Wnt-5a and clinico-pathological variables.

Characteristic	Wnt 5a low (0–1) (n = 146)	Wnt 5a high (2–3) (n = 215)	*p*-value
**Tamoxifen treatment**			
Yes	78 (53%)	101 (47%)	
No	68 (47%)	114 (53%)	
*Missing*	*0 (0%)*	*0 (0%)*	0.2
**Age**			
<45 years	86 (59%)	99 (46%)	
≥45 years	60 (41%)	116 (54%)	
*Missing*	*0 (0%)*	*0 (0%)*	**0.016**
**T size**			
T1	52 (36%)	78 (36%)	
T2	94 (64%)	138 (64%)	
*Missing*	*0 (0%)*	*0 (0%)*	0.9
**Node status**			
N0	41 (28%)	60 (39%)	
N+	104 (71%)	155 (61%)	
*Missing*	*1 (0%)*	*0 (0%)*	0.9
**NHG**			
I	12 (8.5%)	24 (11%)	
II	59 (40%)	89 (41%)	
III	73 (50%)	93 (43%)	
*Missing*	*2 (1.5%)*	*9 (4%)*	0.5
**Ki67**			
<25%	83 (66%)	128 (73%)	
≥25%	43 (34%)	47 (27%)	
*Missing*	*8 (5%)*	*40 (19%)*	0.2
**ER status**			
ER +	84 (60%)	150 (73%)	
ER –	55 (40%)	56 (27%)	
*Missing*	*7 (5%)*	*9 (4%)*	**0.016**
**PR status**			
PR+	85 (61%)	144 (64%)	
PR−	54 (39%)	59 (36%)	
*Missing*	*7 (5%)*	*12 (6%)*	**0.06**
**HER2 status**			
HER2−	123 (91%)	169 (82%)	
HER2+	17 (9%)	38 (18%)	
*Missing*	*6 (4%)*	*8 (4%)*	0.8
**TNBC-phenotype**			
TNBC yes	39 (28%)	38 (18%)	
TNBC no	102 (72%)	169 (82%)	
*Missing*	*0 (0%)*	*8 (4%)*	**0.04**

Data for 361 tumors with two cores.

### Wnt-5a expression is associated with improved recurrence-free survival of the whole cohort

**Figure 2:**
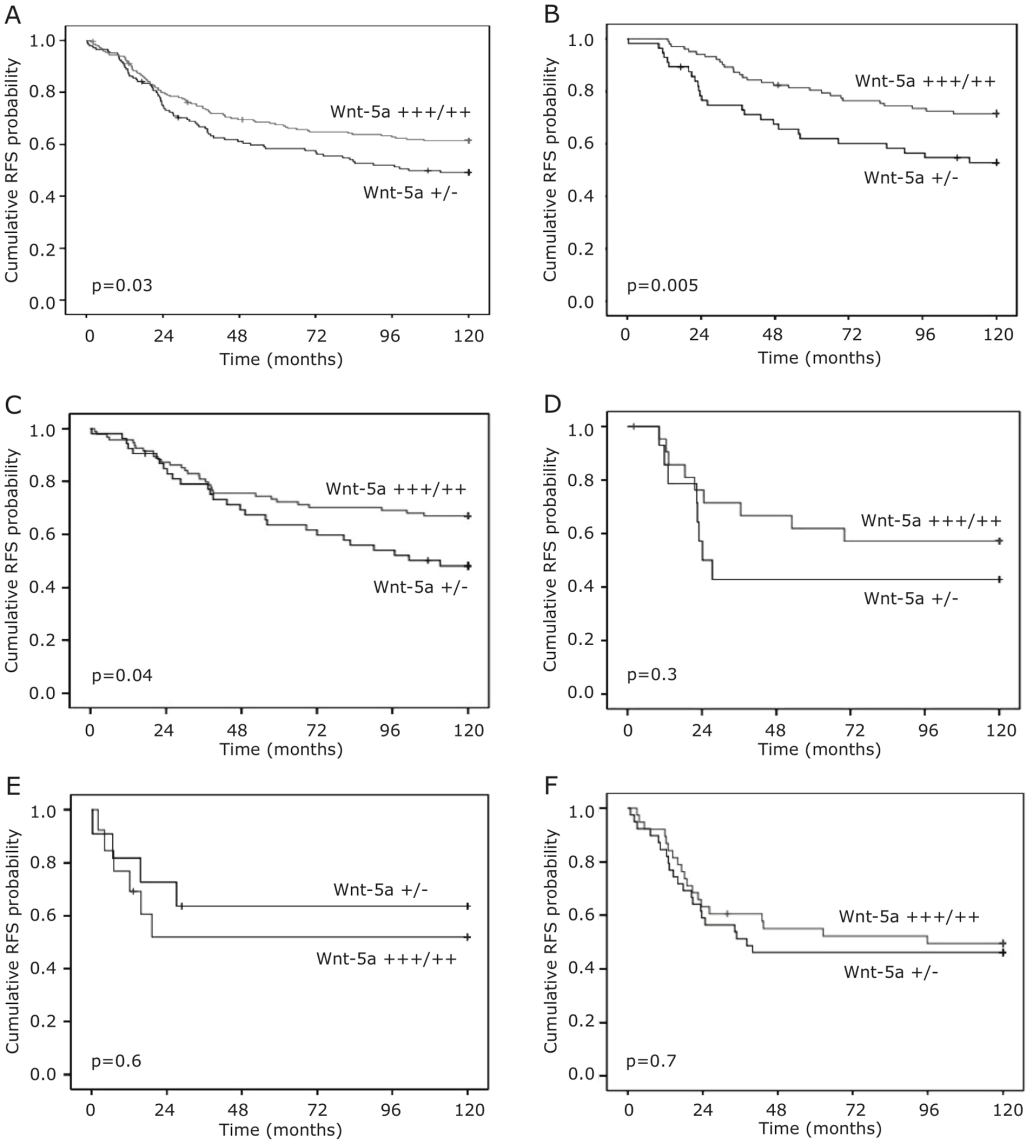
Recurrence-free survival according to Wnt-5a expression. Kaplan–Meier estimates of recurrence free survival according to Wnt-5a status in A. whole cohort, B. ER+ patients. C–F. Kaplan–Meier estimates of recurrence-free survival according to Wnt-5a status stratified for breast cancer subtype. C. Luminal A, D. Luminal B, E. HER2+, and F. TNBC.

For survival analyses, a dichotomised variable defined as low or absent staining versus moderate or strong staining was used. As shown in Figure 2, Wnt-5a protein expression in the whole cohort was associated with an improved Recurrence Free Survival (RFS) (Log Rank test, p = 0.03). We proceeded to perform a Cox regression proportional hazards analysis of RFS to demonstrate estimates of relative risk according to expression of Wnt-5a in univariate and multivariate analyses (Table 2). The multivariate analysis was adjusted for age at diagnosis, ER, PR, tumor size, histological grade, lymph node status and HER2 expression. In the univariate analysis, patients with Wnt-5a-expressing tumors showed an improved RFS compared with patients with Wnt-5a-negative tumors (Hazard Ratio [HR] 0.70; 95% confidence interval [CI] 0.51 to 0.97, p = 0.03). In a multivariate analysis, tumor spread to lymph nodes, large tumor size, and high histological grade were independent markers of poor prognosis. Patients with Wnt-5a-expressing tumors had a favorable prognosis of borderline significance (HR 0.74; 95% CI 0.54–1.02, *p* = 0.06 (Table 2).

**Table 2:** Recurrence-free survival according to Wnt-5a expression.

Whole cohort	Univariate analysis			Multivariate analysis		
Variable	HR	95% CI	p	HR	95% CI	p
**Wnt5a expression**						
Pos *vs* neg	0.7	0.51–0.97	0.03	0.74	0.54–1.02	0.06
**Age**						
<45 yrs *vs* >45 yrs	0.89	0.65–1.22	0.5			
**Node status**						
N+ *vs* N0	1.57	1.07–2.31	0.02	2.00	1.32–3.04	0.001
**Tumor size**						
> 20 mm vs <20 mm	1.38	0.99–1.94	0.06	1.55	1.07–2.25	0.02
**NHG**						
NHG 3 *vs* NHG 1–2	1.41	1.09–1.82	0.009	1.38	1.04–1.83	0.02
**Tamoxifen**						
Yes vs No	0.91	0.66–1.24	0.5			
**HER2 status**						
HER2+ *vs* HER2	1.6	1.02–2.49	0.04	1.32	0.83–2.09	0.2

ER+ tumors	Univariate analysis			Multivariate analysis		
Variable	HR	95% CI	p	HR	95% CI	p
**Wnt5a expression**						
Pos *vs* neg	0.56	0.38–0.85	0.006	0.51	0.33–0.78	0.002
**Age**						
<45 yrs *vs* >45 yrs	0.79	0.53–1.00	0.05	0.93	0.61–1.43	0.7
**Node status**						
N+ *vs* N0	1.51	0.98–2.33	0.06	1.08	0.65–1.80	0.8
**Tumor size**						
> 20 mm *vs* <20 mm	1.26	0.89–1.80	0.2			
**NHG**						
NHG 3 *vs* NHG 1–2	1.49	1.1–2.03	0.01	1.27	0.89–1.89	0.2
**Tamoxifen**						
Yes *vs* No	0.63	0–44–0.89	0.01	0.7	0.45–1.07	0.1
**HER2 status**						
HER2+ *vs* HER2	2.9	1.60–5.24	<0.001	2.84	1.51–5.31	0.001

Cox uni- and multivariate analysis of 10-years recurrence-free survival in patients with tumors evaluated for Wnt-5a in the whole cohort and in ER+ patients only.

### Subset analysis

**Figure 3:**
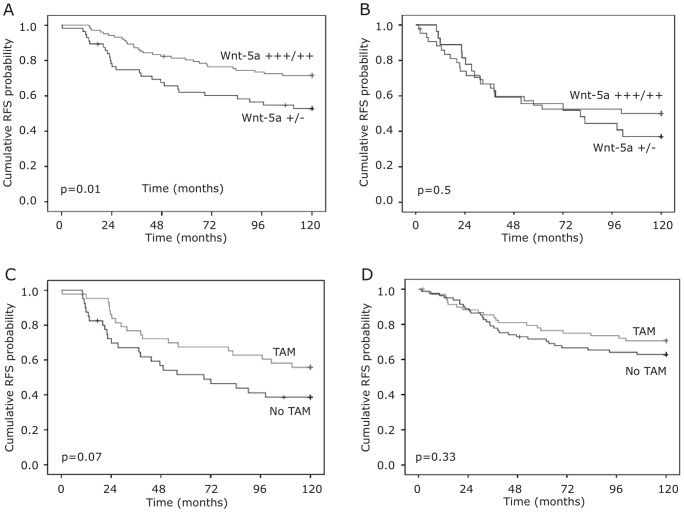
Recurrence-free survival according to Wnt-5a expression and tamoxifen treatment in ER+ patients. Kaplan–Meier estimates of recurrence free survival according to Wnt-5a status in ER+ patients with high/low proliferation rates A. NGH I–II, B. NGH III. Kaplan–Meier estimates of recurrence free survival according to tamoxifen treatment in C. Wnt-5a −/++, and D. Wnt-5a ++/+++ patients.

When stratifying the cohort according to ER status, subset analysis revealed that the favorable effect on outcome associated with Wnt-5a expression was more pronounced in ER+ tumors. In ER+ patients (n = 235) a significantly decreased risk of recurrence was seen in patients with moderate or strong expression of Wnt-5a (HR 0.56; 95% CI 0.38–0.85, p = 0.006). By multivariate analysis, Wnt-5a remained a statistically significant prognostic marker (HR 0.51; 95% CI 0.33–0.78, p = 0.002) after adjusting for standard prognostic factors including TAM treatment. While neither lymph node status nor tumor grade was significantly associated with outcome in the multivariate analysis, expression of HER2 was independently associated with an increased risk of relapse (HR 2.84; 95% CI 1.51 to 5.31, p = 0.001) (Table 2). In the ER- subgroup, no correlation between Wnt-5a expression and outcome was found (p = 0.95). This was true also when restricting the analysis to patients allocated to no adjuvant treatment (p = 0.8). When analyzing patients according to breast cancer subtypes, we found that Wnt-5a was a robust prognostic marker for luminal A tumors (p = 0.04; n = 161) but not for luminal B, HER2+ or triple-negative tumors (Figure 2). In an exploratory analysis including patients with ER+ disease divided according to histological appearance in low (NHG I–II), and high (NHG III) grade tumors, the prognostic effect of Wnt-5a expression was restricted to NHG I–II tumors, (p = 0.01) (Figure 3A–B). Since the luminal A subgroup encompasses tumors with low proliferation indices, with all likelihood corresponding to NHG I and II tumors, this latter finding is not unexpected and supports the result of the subtype analysis. VEGF-A expression did not add any prognostic information in the whole cohort, nor in the ER+ subset (Table 2). When categorizing tumors according VEGF-A expression and Wnt-5a expression into four groups (Wnt-5a low/VEGF-A high, Wnt-5a low/VEGF-A low, Wnt-5a high/VEGF-A high, Wnt-a5a high/VEGF-A low), we were not able to find any prognostic information by adding VEGF-A status to the information obtained by Wnt-5a (*p* = 0.17 log rank test).

### Wnt-5a expression does not predict response to treatment with Tamoxifen

We further explored whether Wnt-5a expression levels could be used as a predictor of response to TAM treatment in patients with ER+ disease. Hence, we explored the ER+ cohort in two strata according to Wnt-5a status; Wnt-5a absent/low and Wnt5-a moderate/high. Patients with ER+/Wnt-5a low tumors (n = 93) had a borderline beneficial effect of TAM in terms of RFS (HR 0.6 (95% CI 0.3–1.0) p = 0.07), but there was no significant effect of TAM in ER+/Wnt5a high tumors (n = 163) (HR 0.7 (95% CI 0.4–1.3) p = 0.3) (figure 3C–D). These results suggest that the addition of TAM does not have any significant effect neither in Wnt-5a high nor Wnt-5a low tumors, but the results were further explored in a Cox multivariate analysis including a term of interaction for Wnt-5a (+/−) and TAM (+/−), Wnt-5a and TAM. The Cox multivariate analysis showed that the term of interaction was not significant (HR 1.3 95% CI 0.6 – 2.9, p = 0.6). Thus, despite some earlier indications from in vitro studies, these data show that the Wnt-5a status of the primary tumor does not affect its sensitivity to TAM.

### Wnt-5a inhibits breast cancer cell invasion irrespective of ER expression

**Figure 4:**
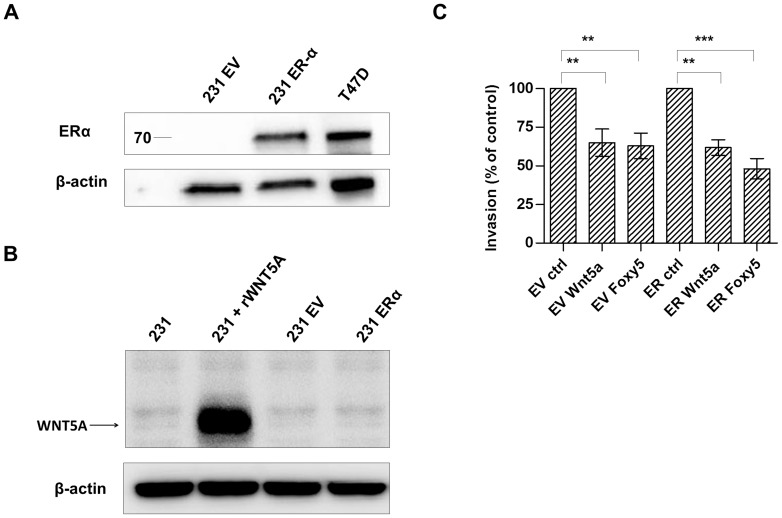
Effect of exogenous ERα expression on the ability of Wnt-5a to decrease breast cancer cell invasion. A. ERα protein expression in MDA-MB-231 cells transfected with pcDNA3 empty vector (231 EV) or ERα expression plasmid (231 ERα). Lysate from the ER-positive breast cancer cell line T47D was included as a positive control. B. Maternal, EV transfected and the ER transfected MDA-MB-231 cells were tested for their expression of Wnt-5a in lanes 1, 3 and 4, respectively. As a positive control recombinant Wnt-5a was added to maternal MDA-MD-231 cell lysate and loaded in lane 2 as a positive control. C. Treatment with recombinant Wnt-5a or the Wnt-5a mimicking peptide Foxy5 significantly reduced the invasive capacity of both ER-negative and ER-positive MDA-MB-231 cells. Error bars, SEM. Paired *t*-test; **, P<0.01, ***, P<0.001.

In previous studies [7], [8] and in the present study we have demonstrated a correlation between the expression of Wnt-5a and ER and/or PR. This resulted in a design of both *in vitro* and *in vivo* experiments where reconstitution of Wnt-5a signaling was only tested in breast cancer cells lacking endogenous expression of both Wnt-5a and ER [16], [17]. The results from these studies revealed that Wnt5a primarily impaired the invasive properties of these cells whereas it only had a minor if any effect on cell proliferation [17]. Based on the present results we deemed it necessary to assess whether Wnt-5a affects breast cancer cell invasion differently in ER-positive compared to ER-negative breast cancer cells. We therefore performed Matrigel invasion assays with control transfected and ER-transfected MDA-MB-231 breast cancer cells. This approach enabled us to compare the possible difference in Wnt-5a-induced effect on cell invasion in ER expressing cells with those lacking ER expression in breast cancer cells with an identical phenotype. Transient ER transfection of ER-negative MDA-MB-231 cells resulted in an ER expression comparable to that of the ER-positive breast cancer cell line T47D (Figure 4A). Control experiments revealed that the Wnt-5a protein was neither expressed in maternal, EV transfected or ER transfected MDA-MB-231 cells (Figure 4B). As shown in figure 4C, stimulation with recombinant Wnt-5a or the Wnt-5a mimicking hexapeptide Foxy5 [16] did significantly decrease the invasive capacity of both ER-negative and ER-expressing cells, demonstrating that the ability of Wnt-5a signaling to impede breast cancer cell invasion is not affected by ER-status.

## Discussion

We have previously shown that loss of Wnt5a is a negative prognostic factor in two unselected cohorts of consecutive patients including few premenopausal women. Thus, the role of Wnt5a in premenopausal breast cancer has until now been unclear. The purpose of the present study was to evaluate whether Wnt5a expression has a unique prognostic value in premenopausal breast cancer patients that could explain the contradictory *in vitro* findings of the effect of Wnt5a on breast cancer cells. Our present results show that Wnt5a expression is a marker of favourable outcome also in premenopausal breast cancer patients and that it is a particularly valuable prognostic marker and thus a possible therapeutic target in pre-menopausal breast cancer patients with ER+ tumors. In contrast, Wnt-5a was not a prognostic factor in premenopausal ER- breast tumors. It is possible, that the Wnt-5a effect in this subgroup is outweighed by the high proliferation rate and other characteristics of bad prognosis characterizing those tumors.

Given the heterogeneity of breast cancer, the present identification of Wnt5a as a potential prognostic biomarker and an indicator of novel future therapies that might aid in further improving the survival of pre menopausal patients with ER+ breast cancers. In premenopausal patients >50% of the patients have ER+ tumors [2]. Generally, ER+ breast cancer has a better prognosis than ER- tumors and ER+ tumors with low proliferating tumors, the luminal A subgroup, are treated by surgery and endocrine therapy alone, whereas the luminal B subgroup will benefit from the addition of chemotherapy. However, up to 40% of ER+ patients will experience tumor recurrence in their lifetime, approximately half of which occur within 5 years after the primary diagnosis [2]. Besides lymph node metastasis, there is as of today no prognostic factor that can be used to identify patients at risk of developing a late recurrence in the ER+ subgroup. The identification of biomarkers for prediction of prognosis would aid in stratification of patients and allow for early selection of optimal therapies. Moreover, it would spare patients with less aggressive tumors a treatment from which they will not benefit. The importance of patient selection for individualized therapy is highlighted in the St Gallen guidelines for the treatment of early breast cancer [3].

We have previously shown *in vitro* that recombinant Wnt-5a and a Wnt-5a-derived peptide impaired migration and invasion of ER-negative breast cancer cells [16], but had no effects on tumor cells proliferation or survival [17]. In the present study we expand these findings by demonstrating that the ability of Wnt-5a to impair breast cancer cell migration is unrelated to the ER status of the tumor cells. Taken together with the present clinical data these findings suggest that the absence or low expression of Wnt-5a in ER+ tumors can cause a more aggressive phenotype by increasing tumor cell invasiveness. The increased tumor cell invasiveness, independent of cell proliferation, caused by loss of Wnt-5a might in such tumors promote breast cancer cell dissemination and the formation of micro-metastases. These micro-metastases can remain dormant for years, eventually forming clinically significant metastases. This is particularly interesting from a therapeutic point of view since a Wnt-5a-derived peptide has been shown to reduce distant metastasis by 70–90% in a breast cancer mouse model [17], suggesting that reconstitution of Wnt-5a signaling in Wnt-5a negative tumors is a promising novel targeted approach to prevent breast cancer metastases.

Since the patients in the study were given adjuvant treatment with TAM or no adjuvant treatment it was possible to compare the effect of TAM in patients with ER+ breast tumors expressing either low levels (−/+) or high (++/+++) levels of the Wnt-5a protein. The data clearly show that in patients with ER+, Wnt-5a expressing tumors, adjuvant treatment with TAM did not improve recurrence-free survival. If this can be explained by the fact that patients with Wnt-5a expressing breast tumors already have a very favorable prognosis remains to be investigated. However, in the ER+ but low Wnt-5a expressing group there was a trend towards increased survival (p = 0.07) with adjuvant TAM treatment.

In the ER+ subgroup there is a particular problem in predicting late recurrence in the low proliferating luminal A subtype. A recent publication suggested a panel of ER, PR, HER2, p53 and Ki67 for improving prognostication of breast cancer [25]. However, for tumors with low proliferation rates, p53 and Ki67 are less likely to provide relevant prognostic information. Our results identify Wnt-5a as a potential independent marker of better prognosis, in premenopausal patients with ER+ breast cancer, including those in the luminal A subgroup. This finding should however be interpreted with caution due to the exploratory nature of the subgroup analysis including a limited number of patients. These results are similar to the previously published data on Wnt-5a as a tumor suppressor and favorable prognostic factor in postmenopausal patients [7], [8]. Consequently, the previous and the present findings are particularly interesting since they suggest that Wnt-5a, a single biomarker determined by immunohistochemistry, could be a useful marker in the clinical setting for both pre- and postmenopausal breast cancer patients.

Taken together, the present study reveal that Wnt5a expression is a marker of favourable outcome also in premenopausal breast cancer patients and that loss of Wnt5a is a particularly valuable prognostic marker and a possible therapeutic target in premenopausal breast cancer patients with ER+ and low proliferative tumors.
